# Altered brain dynamics in post-stroke cognitive and motor dysfunction

**DOI:** 10.3389/fnagi.2025.1640378

**Published:** 2025-08-26

**Authors:** Xiaoying Liu, Guihua Song, Xiaoyun Zhuang, Ying Zhang, Xiaoyang Wang, Yin Qin

**Affiliations:** ^1^Department of Rehabilitation Medicine, 900th Hospital of PLA Joint Logistic Support Force, Fuzhou, Fujian, China; ^2^Fuzong Clinical Medical College of Fujian Medical University, Fuzhou, Fujian, China; ^3^College of Rehabilitation Medicine, Fujian University of Traditional Chinese Medicine, Fuzhou, Fujian, China; ^4^Department of Radiology, 900th Hospital of PLA Joint Logistic Support Force, Fuzhou, Fujian, China

**Keywords:** stroke, motor dysfunction, cognitive and motor dysfunction, hidden markov model, dynamic functional connectivity

## Abstract

**Background:**

Current research is predominantly focused on the single dysfunction after stroke, but the potential changes in brain dynamics of post-stroke cognitive and motor dysfunction (PSCMD) remain unclear, which hinders a deep understanding of its rehabilitation effects. Therefore, the objective is to explore the dynamic brain network characteristics of PSCMD.

**Methods:**

The clinical features and resting-state functional magnetic resonance imaging (rs-fMRI) data were collected from 75 patients with post-stroke motor dysfunction (PSMD), 33 patients with PSCMD, and 35 healthy controls (HCs). Hidden markov model (HMM) was employed for the rs-fMRI data, aiming to identify the repetitive states of brain activity while further assessing the temporal properties and activation patterns in PSCMD. Additionally, the correlation between the HMM state characteristics and clinical scale scores was systematically evaluated.

**Results:**

Five HMM states were ultimately identified. According to the results, PSMD and PSCMD groups showed significant changes in the dynamics of spatiotemporal attributes versus HCs, including fractional occupancy (FO), Lifetime (LT), and transition probability (TP). Furthermore, PSCMD patients exhibited greater FO than PSMD (*p* = 0.006) in state 3. State 3 was mainly characterized by low activation of sensorimotor and higher-order cognitive networks, as well as the high activation of the right prefrontal-parietal network, which may reflect adaptive changes in the brain after PSCMD. Besides, the FO of HMM state 3 exhibited a negative connection with the MoCa score (*r* = −0.389, *p* = 0.025).

**Conclusion:**

An abnormal dynamic brain reorganization pattern could be observed in PSCMD patients. Neuromodulation strategies can be optimized by HMM-derived brain states in the future.

## 1 Introduction

Stroke has become the predominant cause of persistent disability globally (Hilkens et al., 2024). Specifically, survivors tend to manifest as multiple functional impairments, including motor dysfunction, cognitive impairment, speech disorders, and dysphagia (Einstad et al., 2021; Wang et al., 2021; Craig et al., 2022), and over 80% of the patients will experience cognitive and motor concurrent impairments after stroke (Patel et al., 2003). This dual dysfunction has serious effects on patients’ quality of life and results in a huge socioeconomic burden. However, existing studies predominantly focus on single cognitive or motor dysfunction after stroke, while the neural mechanisms underlying post-stroke cognitive and motor dysfunction (PSCMD) are relatively overlooked (Yue et al., 2023; Wang Y. et al., 2024; Pang et al., 2024) which results in a lack of targeted treatment for PSCMD patients, thus exhibiting a negative effect on their rehabilitation outcomes.

Resting-state functional magnetic resonance imaging is extensively employed in the exploration of the neural mechanisms of patients with brain diseases (Viviano and Damoiseaux, 2020; Chen et al., 2021). It has been revealed that intra- and inter-network functional connectivity (FC) of the sensorimotor network (SMN), default mode network (DMN), frontoparietal network (FPN), and salience network (SN) of stroke patients are disrupted, and these FC changes exhibit a strong connection with various dysfunctions after stroke, such as motor and cognitive functions (Cao et al., 2023; Zhang, 2025; Larivière et al., 2018; Vicentini et al., 2021). However, static network analysis methods were employed in all of the studies mentioned above, while the dynamic properties of the network were overlooked (Yue et al., 2023; Pang et al., 2024; Cohen, 2018; Wang et al., 2020; Lu et al., 2024). With the application of a sliding-window dynamic approach, it was found by a recent study that the connections between the visual network and other networks in post-stroke cognitive dysfunction (PSCI) patients were weakened, and they were undetected in static functional connectivity analysis, which underscored the importance of dynamic functional connectivity analysis in the deep exploration of neural mechanisms (Yue et al., 2023). Besides, studies have shown that dynamic brain remodeling has been proven to be a promising approach for creating novel biomarkers for disorders such as cognitive impairment (Xu et al., 2021), schizophrenia (Sun et al., 2019) and epilepsy (Qin et al., 2024). However, there is still a great deal of unknown about how brain networks dynamically remodel after stroke, particularly in PSCMD.

Although sliding window analysis is a commonly used method, it has certain limitations. This approach depends on a fixed window size that requires predetermined size and step increments, and the choice of window size is often somewhat subjective (Vidaurre et al., 2021). Hidden markov model (HMM) has advantages in modeling temporal dependencies and avoiding arbitrary window selection. HMM can accurately capture the transient characteristics of brain state transitions and identify the differences in brain networks of neuropsychiatric patients with its millisecond time resolution (Kottaram et al., 2019; Meer et al., 2020; Javaheripour et al., 2023), thus providing a new perspective for the explanation of the neural mechanisms of neuropsychiatric diseases. For example, Lu et al. (2024) demonstrated that disrupted dynamic reorganization of the DMN might serve as a critical neural factor in cognitive deficits following mild traumatic brain injury. Besides, Zhang et al. (2024) found that frontoparietal structural damage exhibited a strong connection with the decline of activation of the cognitive control network in bipolar disorder. Moreover, Kottaram et al. (2019) demonstrated that the degree of positive symptoms in individuals with schizophrenia exhibited a connection with the enhanced activity in sensory networks and a greater percentage of time spent in states with inactive executive networks (ECN) and DMN. Vidaurre et al. (2017) observed that brain network dynamics are closely related to cognition. Cornblath et al. (2020) found that Temporal sequences of brain activity at rest are modulated by cognitive demands. These findings provided crucial evidence for the deep exploration of the underlying neural mechanisms of PSCMD. Therefore, the application of HMM to PSCMD could be employed in the identification of key biomarkers related to cognitive or motor disorders, thereby providing new insights into the optimization of neural regulation strategies.

In this study, we conducted dynamic brain network analysis of resting-state fMRI data from stroke and healthy controls (HCs) utilizing HMM methodology to identify varying brain states between stroke patients and HCs. We also explored differences in spatiotemporal properties across groups exhibiting distinct dysfunctions after stroke. Based on prior findings, we inferred that stroke patients would experience alterations in state transitions along with specific patterns within their brain networks, alongside potential shared and unique changes in spatiotemporal characteristics between PSCMD and post-stroke motor dysfunction (PSMD) networks.

## 2 Materials and methods

### 2.1 Participant

**FIGURE 1:**
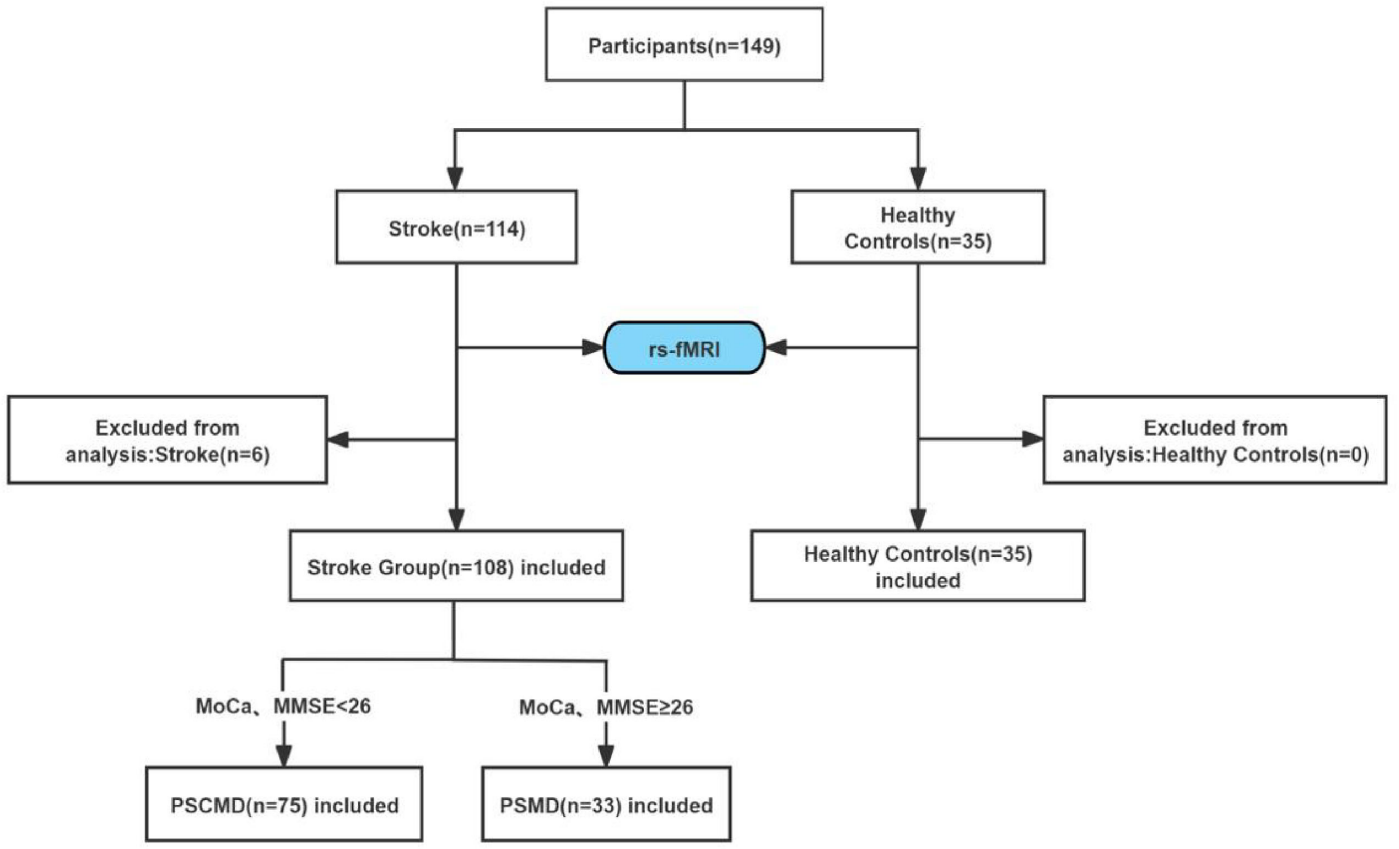
Participant recruitment flowchart.

This research recruited 114 patients with motor dysfunction after stroke. The inclusion criteria were: (1) diagnosis by CT or MRI; (2) first onset of the disease; (3) duration of 2 weeks–3 months; (4) lesions confined to a single hemisphere, primarily involving the basal ganglia and their adjacent areas; (5) age range of 40–75 years old; (6) right-handedness; and (7) stable vital signs and clear consciousness. The exclusion criteria were: (1) history of stroke; (2) history of neurologic or psychiatric disorders, as well as other serious physical diseases; (3) contraindications to MRI examination. Based on the exclusion criteria, four patients were excluded due to previous neurological or mental disorders, and two patients with excessive head movement (translation > 2.5 mm, movement > 2.5°, or mean FD > 0.5 mm) were also excluded. Eventually, 108 patients were incorporated. According to previous research suggestions (Pendlebury et al., 2011; Sachdev et al., 2004), stroke patients were divided into two subgroups: the patients with MMSE score ≥ 26 and MoCA scores ≥ 26were classified as the PSCD group (*n* = 75), while those with MMSE scores < 26 and MoCA scores < 26 were classified as the PSCMD group (*n* = 33). Furthermore, 35 healthy individuals who were matched by age and gender were recruited in this research (Figure 1). This study was approved by the Ethics Committee of the 900 Hospital of the People’s Liberation Army Joint Logistics Force (NO. 2015011). Every participant provided written informed consent.

### 2.2 Clinical assessment

With the application of the Fugl-Meyer assessment scale (FMA), motor function was evaluated in this study (Chen et al., 2022). Besides, daily life abilities were evaluated using the Modified Barthel Index (MBI) (Ohura et al., 2017). The Brief Mental State Examination (MMSE) and the Montreal Cognitive Assessment (MoCa) were employed in the assessment of cognitive function (Folstein et al., 1975; Nasreddine et al., 2005).

### 2.3 MRI acquisition

A 3.0T Siemens Trio scanner (Erlangen, Germany) with a 12-channel phased-array head coil was employed for the whole-brain imaging. Participants were positioned supine, and foam padding was applied to stabilize the head and minimize the motion artifacts. Throughout the scan, subjects were asked to keep their eyes closed and stay awake, maintain relaxed breathing, and avoid voluntary movements. rs-fMRI and detailed 3D T1-weighted structural scans were achieved from all the participants. Gradient-echo planar imaging was employed in the rs-fMRI protocol, and the parameters were set as: repetition time (TR) = 2,000 ms, echo time (TE) = 21 ms, 33 contiguous axial slices (4 mm thickness, 0.8 mm interslice gap), matrix = 64 × 64, field of view = 240 × 240 mm^2^, and a total of 180 volumes were collected over 6 min. 3D T1-weighted parameters were parameterized as follows: TR = 1,900 ms, TE = 2.52 ms, layer thickness = 1 mm, no layer spacing, field of view = 240 × 240 mm^2^, matrix = 256 × 256, number of layers = 176.

### 2.4 Lesion analysis

**FIGURE 2:**
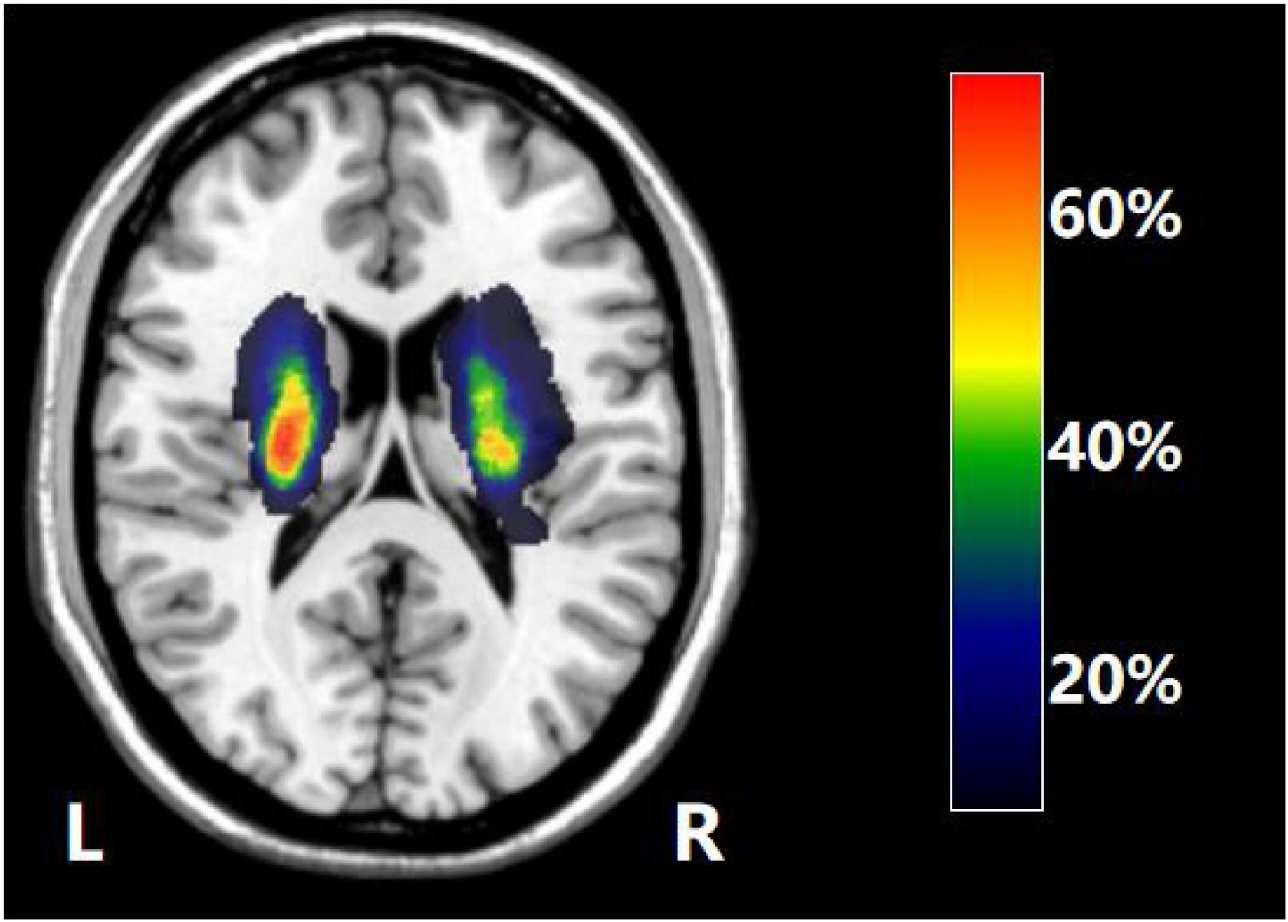
The overlap map of the lesions across all the stroke patients. Color bar indicates the percentage of the lesion overlap.

Lesions were meticulously delineated layer by layer on T1-weighted MR images utilizing MRIcron^1^. Subsequently, the regions of interest (ROIs) were normalized to the MNI spatial template through MR fragment normalization within the SPM8 clinical toolbox. Finally, lesion maps from stroke patients were binarized and overlaid onto T1-weighted templates in the MRIcron software to generate lesion probability maps. We constructed a composite ROI by integrating the normalized ROIs and superimposed this composite ROI onto the T1-weighted template to illustrate the overlapping regions (Figure 2).

### 2.5 Data preprocessing

Before data preprocessing, we flipped the MRI data of patients with left-sided lesions from left to right along the midsagittal line. DPABI^2^ was employed for preprocessing by the following steps: (1) Transform the initial DICOM file into the 4DNifti file format; (2) Remove the first 10 time points, alongside the concurrent slice-time correction and head motion correction on the remaining 170 time points, excluding participants with translation > 2.5 mm, movement > 2.5°, or mean FD > 0.5 mm; (3) Co-register the realigned image with a single 3D T1-weighted image to complete the transformation from a single space to the standard Montreal Institute of Neuroscience space; (4) Smooth on a 6 mm half-height, full-width Gaussian space; (5) Remove noise by regression including Friston’s 24 motor parameters, white matter, cerebrospinal fluid, and global brain signals; (6) Filter in the frequency range of 0.01–0.10 Hz to mitigate the interference of high-frequency noise and low-frequency drift on the images.

### 2.6 Hidden markov modeling

**FIGURE 3:**
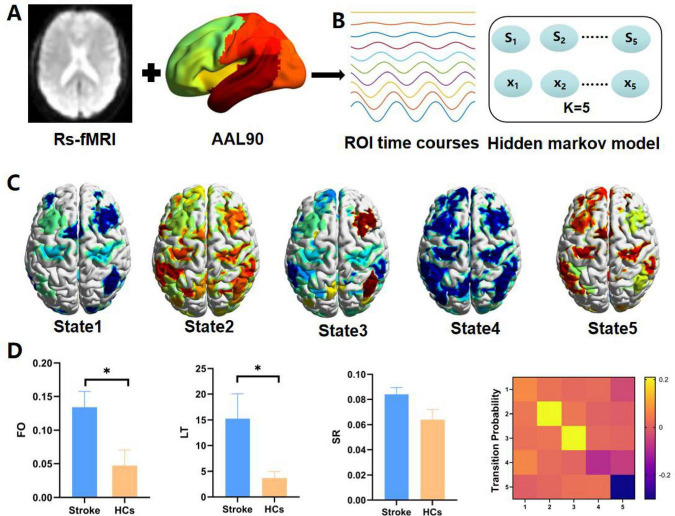
**(A)** The brain regions of each subject were segmented into 90 regions of interest with AAL mapping. **(B)** Time-series data, including 90 areas and 170 time points were collected for all participants. **(C)** Five hidden markov model (HMM) states were identified. **(D)** Four dynamic state metrics [including fractional occupancy (FO), lifetime (LT), switch rate (SR), and transition probability (TP)] were evaluated. * represents *p* < 0.05.

Hidden markov model is a markov process with the incorporation of hidden states. The changes in brain activity over time scales can be explained by a limited number of hidden states. Firstly, the AAL90 brain atlas was employed as the later observation sequence in the extraction of the time series of the subjects after preprocessing. Secondly, 90 ROIs × 170 time-point spatio-temporal data were generated for the construction of HMM. Subsequently, the HMM-MAR toolbox in MATLAB^3^ was used to define states through multivariate Gaussian distributions (Vidaurre et al., 2017). Iterative calculations ranging from 2 to 15 states were conducted following a prior study (Moretto et al., 2022). Finally, the number of different HMM states was evaluated by summarizing statistical indicators, which were composed of the minimum free energy and the median fraction occupancy. It was found that the HMM reached the minimum free energy state when the number of states was 5, and 5 HMM states were selected for subsequent studies (Figures 3A–C).

### 2.7 Temporal properties of HMM

The metrics reflecting temporal brain dynamics were examined: (1) FO: the ratio of time that each subject spent in a specific state. (2) LT: LT is the stretch that a state lingers before shifting into another, which could reflect the stability of the state; (3) Switch rate (SR): it is defined as the frequency of transitions between different states, which could indicate the speed of dynamic changes; (4) TP is the key factor of HMM, which represents the likelihood of changing from a state to another (Figure 3D).

### 2.8 Statistical analysis

The SPSS 23.0 software (SPSS Inc., Chicago, Illinois, United States) was utilized in this study. Continuous variables were expressed as mean ± standard deviation (SD), while categorical variables were presented as counts. The Shapiro-Wilk test was employed in the assessment of the normality of the data. Continuous variables were assessed by independent *t*-tests or one-way analysis of variance (ANOVA), while categorical variables were analyzed with chi-square tests. A two-tailed *t*-test was applied in the comparison of FO, LT, and SR of HMM status in stroke and HCs, as well as PSMD and PSCMD patients. False discovery rate (FDR) correction was applied to multiple comparisons. *p* < 0.05 threshold was considered to indicate statistical significance.

A non-parametric permutation test was utilized to compare HMM state transition probabilities between stroke patients and HCs, as well as PSMD and PSCMD, with the involvement of 5,000 permutations. Moreover, Spearman correlation analysis was employed in the evaluation of the connections between HMM state-time attributes and FMA and MMSE scores. *p* < 0.05 threshold was considered to indicate statistical significance.

### 2.9 Verification analysis

To validate the robustness of the results obtained from the HMM approach, we perform a validation analysis using the sliding window analysis method and K-means clustering framework. Firstly, the dynamic functional network connectivity (FNC) was computed using a sliding time window approach with the window size set to 22 TR (44s) and a step size of 1 TR. Secondly, a K-means clustering algorithm was applied to the windowed FNC matrix to extract the same number of states as in the HMM model (*K* = 5). Finally, the temporal properties of the dynamic FNC states were extracted to assess the consistency of the population trends.

## 3 Results

### 3.1 Demographic and clinical data

A total of 108 ischemic stroke patients and 35 HCs were included in this research. Regarding the aspects of age and gender, no significant differences were observed between the stroke patients and HCs. In stroke subgroup analysis, the MoCA scores and MMSE scores were significantly different between PSMD and PSCMD (*p* < 0.05), whereas no differences could be found in the aspects of stroke duration, type, lesion hemisphere, FMA, and MBI (Table 1).

**TABLE 1:** Demographic and clinical characteristics.

Variable	PSMD (*n* = 75)	PSCMD (*n* = 33)	HCs (*n* = 35)	*P*-value
Age, y, mean (SD)	56.95 ± 1.098	60.21 ± 1.675	57.69 ± 1.321	0.234
Sex, male, *n* (%)	55 (73%)	18 (55%)	20 (57%)	0.090
Stroke duration, *d*, mean (SD)	33.91 ± 23.29	33.85 ± 20.56	NA	0.990
Stroke type, ischemia, *n* (%)	71 (95%)	30 (91%)	NA	0.456
Lesion hemisphere, left, *n* (%)	42 (56%)	17 (52%)	NA	0.666
FMA, mean (SD)	59.71 ± 25.14	52.48 ± 23.42	NA	0.163
Barthel, mean (SD)	69.64 ± 19.92	62.24 ± 22.46	NA	0.090
MMSE, mean (SD)	28.89 ± 1.09	21.91 ± 4.52	NA	<0.001
MoCa, mean (SD)	28.21 ± 1.11	17.30 ± 6.38	NA	<0.001

HCs, healthy controls; PSMD, post-stroke motor dysfunction; PSCMD, post-stroke cognitive and motor dysfunction; M ± SD, mean ± standard deviation.

### 3.2 Average functional activity of HMM states

As shown in Figure 3C, State1 is mainly a decrease in activation for most regions, but its average functional activity is relatively higher than that of state 4. State2 is mainly a relative increase in activation for most regions, but its average functional activity is relatively lower than state 5. State 3 is mainly characterized by relatively decreased activation in most areas, but increased activation in the right frontal_mid, right frontal_mid_orb, right temporal_sup, right inferior parietal lobule, right coronal gyrus, and right anterior cuneus. State4 is primarily a decrease in overall regional activation. State5 is primarily an increase in overall regional activation.

### 3.3 Dynamics of each HMM state

**FIGURE 4:**
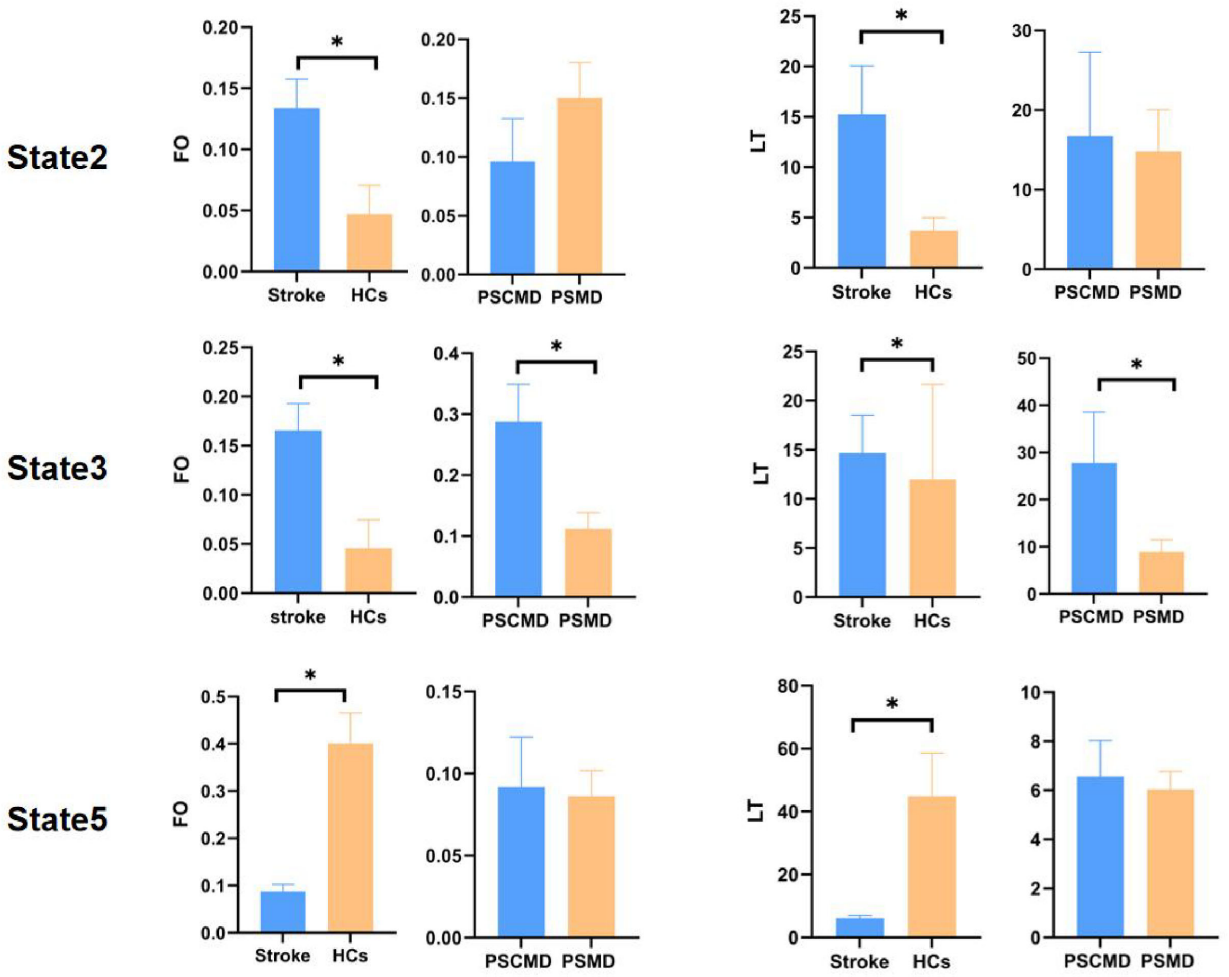
Fractional occupancy (FO) and lifetime (LT) in different hidden markov model (HMM) states of the stroke and healthy controls (HCs), as well as post-stroke motor dysfunction (PSMD) and post-stroke cognitive and motor dysfunction (PSCMD). Blue represents stroke patients or PSCMD patients, and yellow represents HCs or PSMD patients. *p < 0.05.

Compared with HCs, stroke patients exhibited noticeably higher FO (*t* = 2.603, *p* = 0.018, FDR-corrected) and LT (*t* = 2.255, *p* = 0.040, FDR-corrected) in State 2, as well as FO (*t* = 3.025, *p* = 0.008, FDR-corrected) and LT (*t* = 2.903, *p* = 0.010, FDR-corrected) in State 3; whereas FO (*t* = −4.732, *p* = 0.0001, FDR-corrected) and LT (*t* = −4.175, *p* = 0.0002, FDR-corrected) in State 5 exhibited a significant decreasing trend. Furthermore, it was revealed by subgroup analyses that PSCMD patients showed significantly higher FO (*t* = 3.096, *p* = 0.006, FDR-corrected) and LT (*t* = 2.332, *p* = 0.022, FDR-corrected) in State 3 than PSMD patients (Figure 4).

### 3.4 Transition patterns between HMM states

**FIGURE 5:**
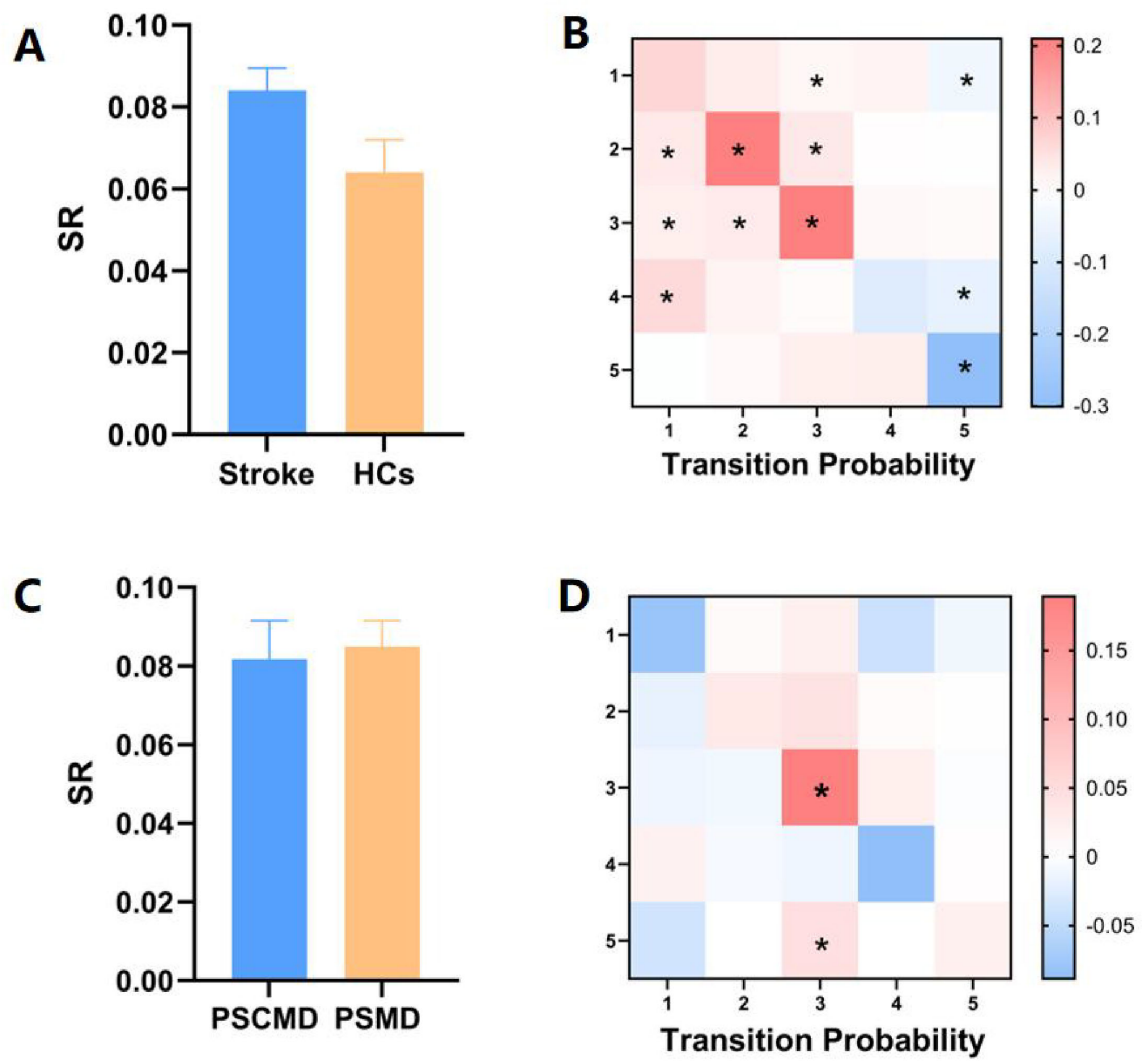
Variations in state transitions between stroke patients and healthy controls (HCs), as well as between post-stroke motor dysfunction (PSMD) and post-stroke cognitive and motor dysfunction (PSCMD) subgroups. **(A)** Differences in switch rate (SR) between stroke patients and HCs; **(B)** notable shifts in transition probability (TP) between the two groups. Red color indicates a marked increase in stroke patients relative to HCs, while blue color denotes a significant decline; **(C)** contrasts in SR between PSMD and PSCMD patients; **(D)** distinct changes in TP between PSMD and PSCMD patients. Red color indicates a marked increase in PSCMD patients versus PSMD patients, and blue color indicates a significant decrease in PSCMD patients versus PSMD patients. *p < 0.05.

According to the comparison of the transfer patterns of HMM states, no significant difference in SR could be observed (*p* > 0.05) (Figure 5A), which could suggest that stroke patients exhibited stable network dynamics (similar to HCs) during resting-state scanning. However, the TP of HMM states varied between the two groups (Figure 5B). Specifically, compared with HCs, stroke patients exhibited a significantly higher transition probability for state 1 to state 3, state 2 to states 1–3, state 3 to states 1–3, and state 4 to state 1 (state 1 to 3: *p* = 0.011; state 2 to 1: *p* = 0.031; state 2 to 2: *p* = 0.010; state 2 to 3: *p* = 0.001; state 3 to 1: *p* = 0.040; state 3 to 2: *p* = 0.007; state 3 to 3: *p* = 0.011; state 4 to 1: *p* = 0.025). Besides, stroke patients exhibited a significantly lower probability of transitioning from HMM states 1, 4, and 5 to state 5 (state 1 to 5: *p* = 0.002; state 4 to 5: *p* = 0.001; state 5 to 5: *p* = 0.00001). Overall, it could be implied by these results that stroke patients exhibited a noteworthy pattern of aberrant transitions between HMM states.

In the stroke subgroup, no discernible variation was observed in SR among the HMM statuses (Figure 5C), while significant differences could be found in TP between the PSMD and PSCMD groups. Compared with PSMD, patients with PSCMD show significantly higher transition probabilities from state 3 and state 5 to state 3 (state 3 to 3: *p* = 0.013; state 5 to 3: *p* = 0.019) (Figure 5D).

### 3.5 Brain activation maps of states

**FIGURE 6:**
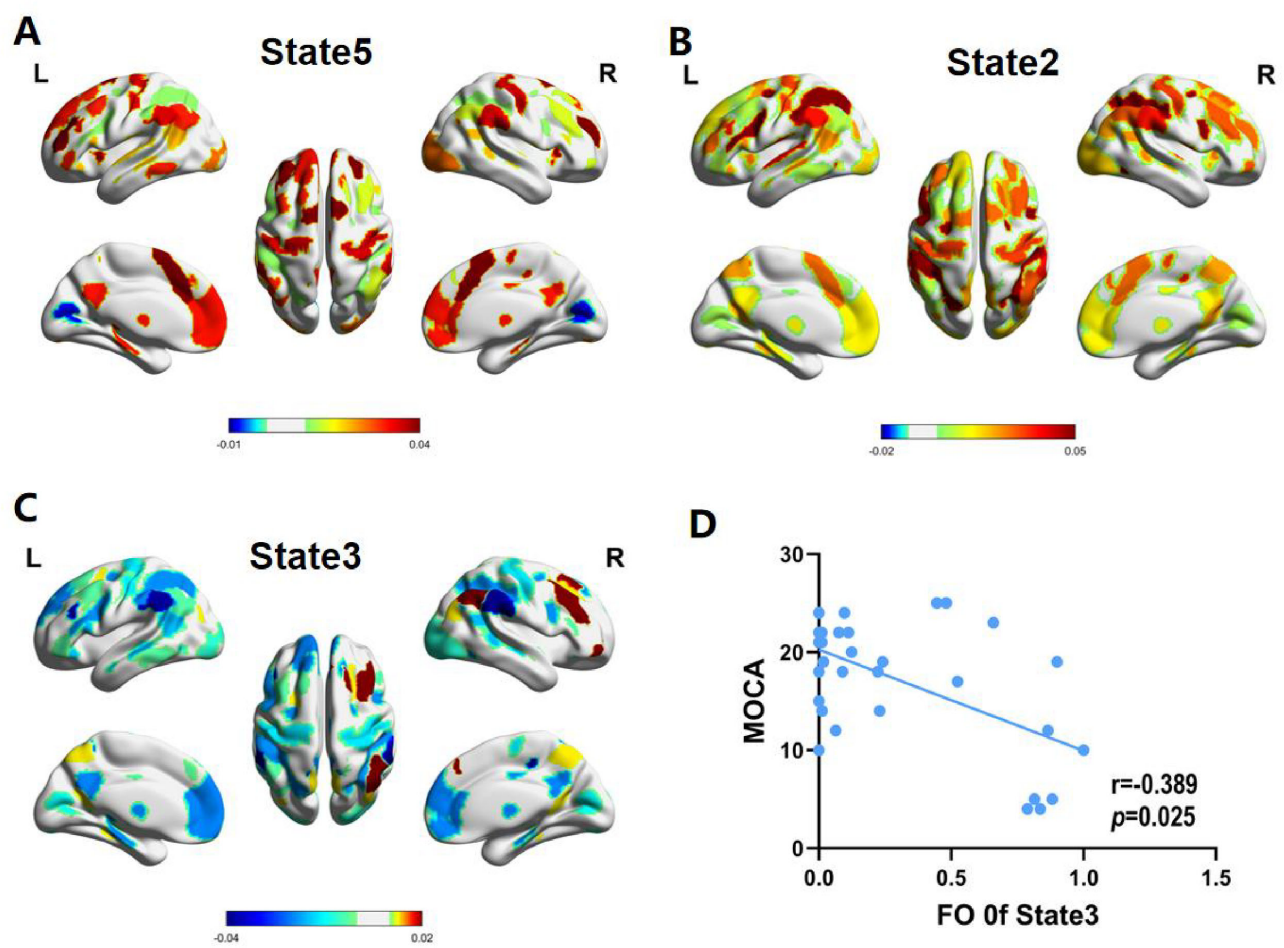
Spatial activation patterns of hidden markov model (HMM)-derived brain states. **(A)** Average activation patterns of state 5 mainly triggered by healthy controls (HCs). **(B)** Average activation patterns of state 2 mainly triggered by post-stroke motor dysfunction (PSMD). **(C)** Average activation patterns of state 3 mainly triggered by post-stroke cognitive and motor dysfunction (PSCMD). **(D)** Fractional occupancy (FO) of HMM state 3 exhibited a negative connection with the Montreal Cognitive Assessment (MoCa) scores.

The spatial activation patterns of large-scale whole-brain network states were investigated in this study. Compared to stroke patients, HCs were mainly characterized by state 5 (Figure 6A). State 5 showed that regions with enhanced activation were mainly located in the precentral gyrus, the postcentral gyrus, the supplementary motor area, the superior frontal gyrus, the middle frontal gyrus, the precuneus, the subparietal lobule, the subcortical regions (thalamus, caudate nucleus, and nucleus of the bean-shell), and the hippocampus. Conversely, areas with weakened brain activation were predominantly in the cuneate lobes.

According to the results, stroke patients were predominantly in states 2 and 3 (Figures 6B, C). Specifically, the brain regions with enhanced activation in state 2 were mainly composed of the precentral gyrus, supplementary motor area, superior frontal gyrus, middle frontal gyrus, precuneus, subcortical regions, middle occipital gyrus and inferior occipital gyrus, and cuneus, while the activation levels were significantly lower than state 5. The regions with weakened activation in state 3 were mainly in the middle frontal gyrus, superior frontal gyrus, precentral gyrus, left supplementary motor area, subcortical regions, middle temporal gyrus, inferior temporal gyrus, angular gyrus, middle occipital gyrus and inferior occipital gyrus, and insula, whereas those with enhanced activation were predominantly in the right superior frontal gyrus, right middle frontal gyrus, right inferior parietal lobule, right angular gyrus, and right precuneus. Regarding the stroke subgroup analysis, PSMD patients were mainly dominated by state 2, while PSCMD patients were mainly dominated by state 3.

### 3.6 Correlation analysis

A negative correlation could be found between FO and severity of cognitive impairment in state 3 of PSCMD patients (*r* = −0.389, *p* = 0.025) (Figure 6D), while the temporal dynamic characteristics of other states exhibited no significant connections with motor or cognitive functions.

### 3.7 Validate analysis results

Sliding window analysis and K-mean clustering were used to obtain five states, and between-group differences in the temporal attributes of these states were calculated. We found that stroke patients had higher FO in state 3 (*t* = 2.933, *P* = 0.010, FDR-corrected), but shorter FO in state 5 (*t* = −4.732, *P* = 0.0001, FDR-corrected) compared with HCs. The comparison between stroke subgroups showed that the FO of PSCMI patients in state 3 was higher than that of PSMD patients (*t* = 2.436, *P* = 0.017, FDR-corrected). In addition, there was no difference in number of transitions between stroke and HCs group as well as the stroke subgroup in the analysis (*P* > 0.05). These are similar to the HMM results. However, we did not find a difference between stroke and HCs in state 2 in the validation analysis (Supplementary Figure 1).

## 4 Discussion

According to the evaluation of fMRI data by HMM, five states that were characterized by average functional activity and functional connectivity were identified. It was found that stroke patients exhibited abnormal connectivity and activation of multiple brain networks, which could suggest the widespread impairment of neural network integration after stroke. Besides, further subgroup analyses revealed that patients with PSCMD exhibited low activation of sensorimotor networks and higher-order cognitive networks, while the right prefrontal parietal network was highly activated. These findings suggested heterogeneity in the network dynamics of stroke patients with different functional disabilities, and HMM-derived brain states could be employed for the optimization of future neural regulation strategies.

According to recent research, fMRI can be utilized in the detection of the dynamic reconfiguration of extensive brain activity after stroke (Mao et al., 2025; Zhang et al., 2025; Wang X. et al., 2024). Notably, it was found that the LT and FO of the stroke group were significantly lower in state 5, which was characterized by strong intra- and inter-network connections, whereas the FO was significantly higher in state 3, which was characterized by weaker intra- and inter-network connection strengths. This could suggest that stroke patients were more prone to staying in a low integration state, which was consistent with prior studies based on the sliding window approach (Wang et al., 2020; Bonkhoff et al., 2020). Importantly, compared with PSMD, PSCMD exhibited higher FO in State 3, which was characterized by diminished functional connectivity between the DMN and the FPN. Functionally, the FPN was essential for cognitive control and task switching, coordinating the allocation of attentional resources, while the DMN exhibited a strong connection with the primary sensory processing and higher-order cognitive integration. Previous studies have demonstrated FPN in subcortical stroke patients, alongside the FC reduction in the network of the anterior DMN (Wang et al., 2014). Additionally, patients with PSCI showed weakened local functional connectivity in cognitive networks, including the DMN, and orbitofrontal cortex (Miao et al., 2021). Collectively, it could be suggested by these findings that the elevated FO in State 3 among PSCMD patients may involve impaired frontal-parietal integration and misallocation of attentional resources, reflecting diminished integration efficiency of higher-order cognitive networks. In addition, MMSE scores in stroke patients exhibited a significantly negative connection with FO in state 3, indicating that an increased proportion of time spent by patients in this particular state correlates with more pronounced impairments in cognitive function.

The brain is a complex and dynamic system (van der Horn et al., 2017; Ahrends et al., 2022), which can support cognitive and motor regulatory functions by switching between different brain states. By analyzing the switching rate of HMM states, we found that there was no significant difference in SR between stroke patients and HCs, but there was difference in TP between the two groups. The lack of significant difference in SR between the two groups suggests that the overall frequency of brain state transitions after stroke has a certain degree of stability, whereas the difference in TP reflects the reorganization of transfer paths between specific states in stroke patients. This may be related to the dynamic balance mechanism of the brain. Stroke patients may maintain overall stability through healthy-side compensation, but damaged networks exhibit altered state preferences. Notably, PSCMD patients were more prone to shifting to the weakly connected state 3 and staying in this state for a long time, which might be attributed to the weakened cognitive flexibility, and this should be further investigated in the future to explore its application value as a potential biomarker for PSCMD.

Based on the spatial activation map analysis of comprehensive cerebral network states, HCs were mainly characterized by state 5, showing synergistic positive activation of SMN, DMN, and FPN. It could be suggested that HCs exhibited efficient integration of multiple networks in the resting state. Conversely, a marked reduction in the activation of several brain networks was observed in stroke patients, which was consistent with prior research findings (Zhang, 2025; Chen et al., 2025; Ding et al., 2018). However, it was found that PSMD patients exhibited enhanced visual network (VN) activation versus HCs. It was indicated that abnormal functional connectivity of the VN and diminished cross-network connectivity might serve as the explanation for the pathomechanism of the defective motor-visual attentional integration after stroke, which was critical for the recovery of sensorimotor functions by driving neuroplasticity (Zappasodi et al., 2017; Zhao et al., 2018). Based on the above findings, it was inferred that VN hyperactivation in PSMD patients might reflect a compensatory reorganization pattern, which was based on the enhancement of visuomotor integration to partially compensate for impaired sensorimotor pathways (Zhao et al., 2018). Notably, PSCMD exhibited high activation of the right prefrontal-parietal networks, and this hyperactivation might be attributed to the network reorganization after brain microstructural damage. It has been indicated that the application of stimuli into the active large-scale targeted-network could enhance the cortical excitability, and it is more favorable for functional recovery (Sack et al., 2024). Therefore, the present findings not only have significant contributions to the identification of neuromarkers of PSCMD, but also have the potential to serve as “targets” for the neuromodulation of tDCS or TMS in PSCMD patients.

## 5 Limitations and future directions

However, several limitations still exist in this study. First, there is an imbalance in the sample size between stroke groups and HCs, which may exert a negative effect on the statistical validity of the group difference analysis. Therefore, the sample size needs to be enlarged in the future, in order to validate the broad applicability of dynamic features. Second, only the cross-sectional data were analyzed in the current study, which relatively limited the causal inference of the connections between the dynamic evolution of brain networks and clinical prognosis. Future studies should incorporate longitudinal data tracking neuroplasticity trajectories in stroke patients. Explore the integration of HMM or dFNC-derived dynamic metrics into clinical classifiers or prognostic models to assess their translational potential in identifying and stratifying PSCMD patients. Third, it’s challenging to figure out the best number of HMM states (Stevner et al., 2019; Hutchison et al., 2013; Hindriks et al., 2016). Although *k* = 5 is identified as the optimal solution for the current data, transient dynamic features specific to stroke may be overlooked. Overall, the increase in state numbers could improve the temporal resolution, while it was necessary to keep a balance between the model complexity and interpretability. Finally, differences in lesion locations, disease duration, and degree of impairment in stroke patients make our patient group highly heterogeneous, which may influence our interpretation. In the future, more detailed functional evaluations should be incorporated to refine each subgroups for further research.

## 6 Conclusion

Post-stroke patients exhibited abnormal activation patterns and broken functional connectivity across various brain networks, and specific changes were found among different subgroups. Notably, PSCMD patients demonstrated hypoactivation in extensive sensorimotor networks and higher-order cognitive networks, but they concurrently exhibited hyperactivation in the right prefrontal-parietal networks. Overall, these findings provide a basis for the development of brain-state-dependent stimulation strategies in neuromodulation interventions for stroke rehabilitation.

## Data Availability

The raw data supporting the conclusions of this article will be made available by the authors, without undue reservation.

## Footnotes

1
http://www.mccauslandcenter.sc.edu/mricro/mricron

2
http://rfmri.Org/dpab

3
https://github.com/OHBA-analysis/HMM-MAR
