# Supplementary material for: Altered brain dynamics in post-stroke cognitive and motor dysfunction
Source: Front Aging Neurosci. 2025 Aug 26;17:1640378. doi: 10.3389/fnagi.2025.1640378 (PMC12417414; doi:10.3389/fnagi.2025.1640378)

**Figure S1:**Validate analysis results.FO and number of transitions in different HMM states of the stroke and HCs, as well as PSMD and PSCMD. Blue represents stroke patients or PSCMD patients, and yellow represents HCs or PSMD patients. **p*< 0.05.


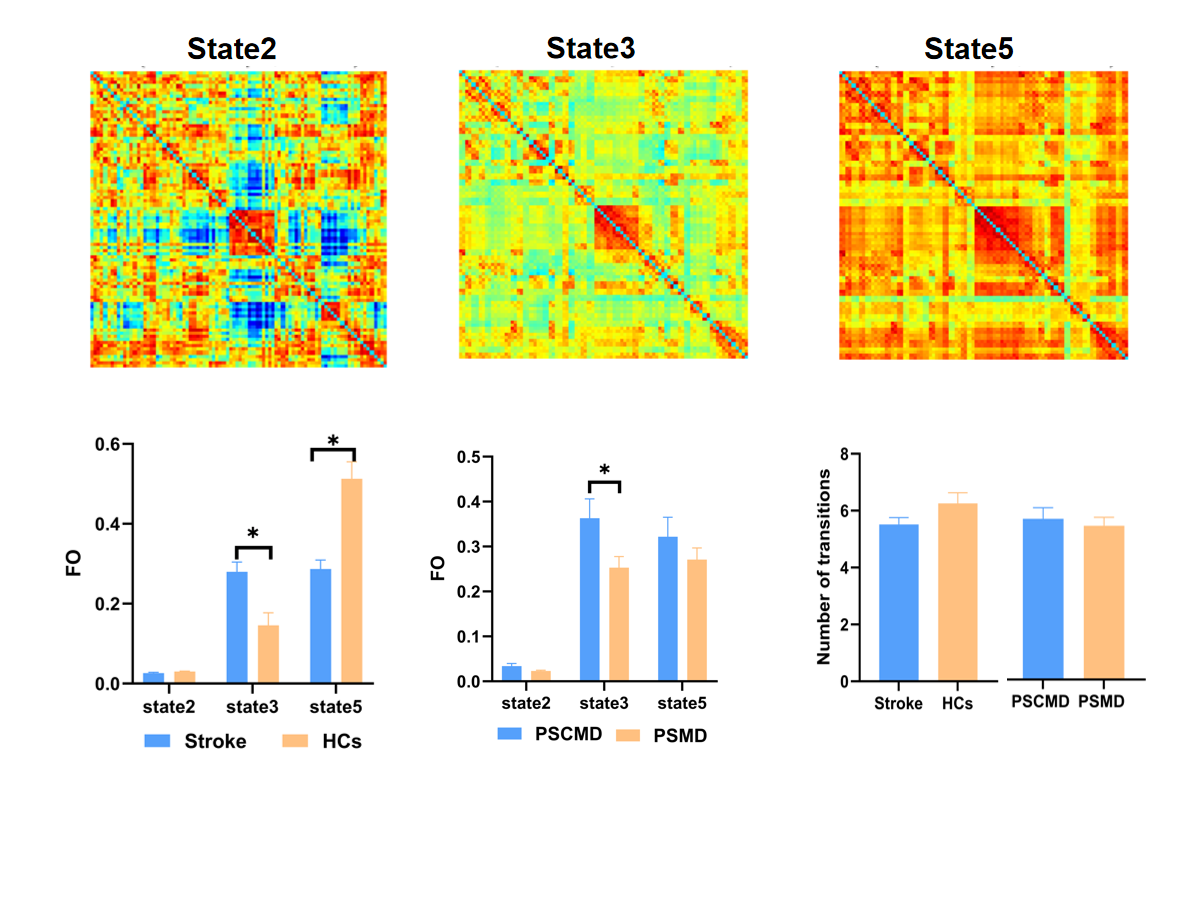

Supplement: Supplementary file 1 [file Table_1.docx]
